# Nonsurgical treatment for a severe anterior and lateral open bite and multiple congenitally missing teeth: a case report with 4-year follow-up

**DOI:** 10.1590/2177-6709.22.6.074-085.oar

**Published:** 2017

**Authors:** Ana Paula Abdo Quintão, Livia Kelly Ferraz Nunes, Renato Barcellos Rédua, Ione Helena Portela Brunharo, Catia Cardoso Abdo Quintão

**Affiliations:** 1 Private practice (Juiz de Fora/MG, Brazil).; 2 Universidade do Estado do Rio de Janeiro, Programa de Pós-graduação em Ortodontia (Rio de Janeiro/RJ, Brazil).; 3 Escola Superior São Francisco de Assis, Faculdade de Odontologia, Disciplina de Clínica Integrada Infantil (Santa Tereza/ES, Brazil).; 4 Private practice (Rio de Janeiro/RJ, Brasil).

**Keywords:** Open bite, Anodontia, Orthodontic appliances

## Abstract

This case report describes the treatment of a severe anterior and lateral open bite combined with multiple congenitally missing teeth. A 10-year-old girl presented with an open gonial angle, absence of lip sealing, and soft tissue pogonion retrusion. She had an open bite of 8.5 mm, agenesis of the upper right and left lateral incisors and the upper left first premolar, and transverse maxillary deficiency. Nonsurgical treatment was planned aiming at controlling the vertical pattern, establishing the correct overbite, and closing the spaces on the upper arch, to provide satisfactory occlusion and facial and dental esthetics.

## RESUMO

O presente caso clínico descreve o tratamento de uma mordida aberta anterior e lateral associada à ausência congênita de dentes permanentes. Paciente com 10 anos de idade, apresentava ângulo goníaco aberto, ausência de selamento labial passivo e retrusão do pogônio mole. Além disso, foi diagnosticada uma mordida aberta de 8,5 mm, agenesia de incisivos laterais superiores direito e esquerdo e de primeiro pré-molar superior esquerdo, além de deficiência transversa da maxila. O planejamento do caso envolveu um tratamento não cirúrgico, com controle vertical do crescimento, obtenção de correta sobremordida e fechamento dos espaços superiores. O caso foi finalizado com uma boa intercuspidação, contemplando a estética facial e dentária.

## INTRODUCTION

Orthodontists find it difficult to determine the prognoses of open bite malocclusions because they develop due to many etiologic factors and have a moderate rate of relapse.^1^ ^-^ ^4^

Several issues, such as mouth-breathing, sucking habits, tongue-thrusting, vertical maxillary excess, vertical growth skeletal patterns, and abnormalities in dental eruption can - individually or collectively - contribute to a severe open bite.^5^ ^-^ ^7^

Mechanical treatment options for open bite are limited, and orthognathic surgery is indicated in adult patients with severe open bite and unesthetic facial proportions. The search for effective nonsurgical treatment modalities for less severe growth problems continues.^2^

Missing or malformed teeth are of great concern to orthodontists. The maxillary lateral incisor is the second most common congenitally absent tooth. Replacement treatments for missing lateral incisors include canine substitution, tooth-supported restoration, single-tooth implantation, and autogenous tooth transplantation.^8-11^ Appropriate therapy is selected based on several factors, including the degree of malocclusion, specific space requirements, the tooth-size relationship, and the size and shape of the canines. The ideal treatment should optimally meet both individual esthetic and functional requirements.^12^ ^-^ ^14^

In this case report, we present the nonsurgical treatment of a patient with a vertical growth pattern, an 8.5-mm anterior open bite, a posterior crossbite, and agenesis of the upper right and left lateral incisors, and the upper left first premolar. The patient was treated with a vertical chincup, fixed appliances, and intermaxillary elastics during the pubertal growth spurt.

## DIAGNOSIS AND ETIOLOGY

A 10-year-old female patient in the pubertal growth spurt presented for orthodontic treatment on a private orthodontic practice. She had severe anterior and lateral open bite, posterior crossbite in centric relation (as determined by bilateral manipulation), agenesis of the upper right and left lateral incisors, and the upper left first premolar; with upper and lower left deciduous first molars with ankylosis. There was no previous history of this type of malocclusion in her family, and she had no symptoms of temporomandibular joint disorder.

The patient presented with a history of respiratory allergies, hypertrophied adenoids, mouth-breathing, and atypical swallowing, with the chief complaint of being unable to bite with her front teeth.

**Figure 1:**
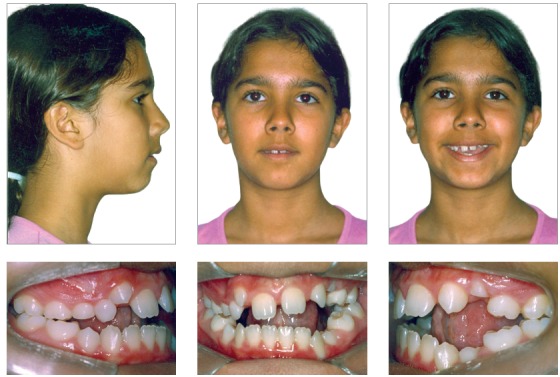
Pretreatment facial and intraoral photographs showing a 8.50-mm negative overbite.

Facial analysis revealed an increase in the size of the lower third of the face, exposure of the upper incisors at rest, thin lips, a slight reversion of the lower lip, and a right-tilted smile line. A convex profile, absence of lip sealing, and soft tissue pogonion retrusion were also observed (Fig 1). 

**Figure 2:**
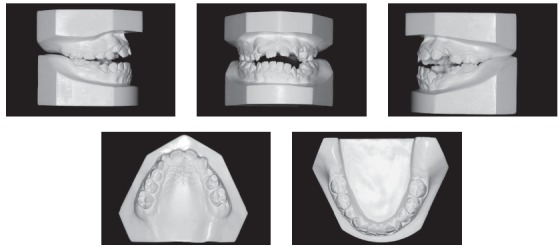
Pretreatment study casts.

The initial intraoral photographs and dental casts revealed a Class I molar relationship with Class II right canines and Class I left canines, an 8.5-mm anterior open bite, a 4-mm left deviation of the lower midline, a crossbite of the upper right deciduous canine and first molar with the lower right first premolar, an upper left deciduous second molar, and a lower left deciduous second molar. The maxillary arch had a triangular shape and included general spaces (Fig 2).

**Figure 3:**
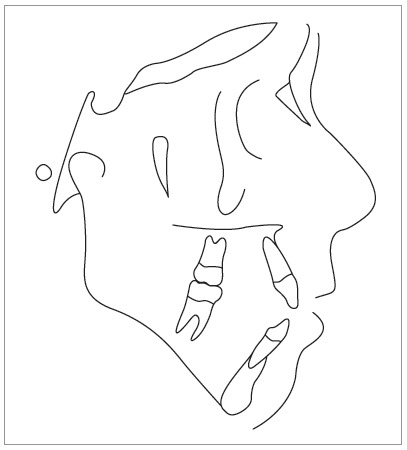
Pretreatment cephalometric tracing.

Cephalometric analysis revealed a convex skeletal profile, an open gonial angle, a narrow and long mandibular symphysis, characteristic of a dolichofacial pattern, a deficient maxillomandibular relationship, well-positioned maxillary incisors, and protruded and buccally tipped lower incisors (Fig 3).

## TREATMENT OBJECTIVES

The main objectives of the first phase of treatment were to control the vertical growth of the face, minimize the anterior open bite, and improve the shape of the upper arch, all in combination with speech therapy.

In the second phase, the objectives were to control the vertical pattern, establish the correct overbite and overjet, close the spaces of the upper arch with forward movement of the upper canines, and to create a Class II relationship for the right and left molars, to provide satisfactory occlusion and facial and dental esthetics^15^.

## TREATMENT ALTERNATIVES

The treatment alternatives included orthopedic treatment followed by orthodontic treatment, orthodontic treatment only, or orthodontic treatment in combination with orthognathic surgery. Moreover, the closing or opening spaces at the points of tooth agenesis could be approached with placement of dental implants or transplants.

Orthognathic surgery was extensively discussed with the patient and her parents, since it was determined that correcting the skeletal discrepancy, achieving the desired facial and dental esthetic change, and establishing an ideal occlusion would all be possible with this surgical approach. However, the parents did not have complaints regarding facial esthetics; the patient’s complete growth prior to the surgery was important. Thus, we selected a nonsurgical treatment.

As the patient presented at the orthodontic clinic at the age of 10 years and was within the pubertal growth spurt, a two-phase treatment plan was selected. The first phase included rapid maxillary expansion with an encapsulated device or the frequent use of a vertical chincup with 500g force/side and the extraction of the ankylosed teeth.

For the second treatment phase, a chincup with vertical hooks (sky hooks) was employed for the forward movement of the upper posterior teeth and the closure of the anterior spaces. Although temporary anchorage devices (TADs) could be used for this type of mechanics, the patient and her parents refused all forms of surgical intervention.

The teeth were aligned using stainless steel archwires that were shaped by bending them to compensate for the anterior teeth. Anterior elastics were then used, and this phase was completed via the compensation of wire bending.

Regarding the agenesis of the upper lateral incisors and premolars, the treatment options included opening spaces for implant placement, tooth-supported restoration, or autogenous tooth transplantation. They chose closure of spaces because the patient was young and would have to wait until her growth was completed for implant placement. In addition, only natural teeth were to be kept, without future surgeries.

## TREATMENT PLANNING

The patient had no facial complaints, with continuing facial growth; thus, a nonsurgical treatment was chosen. After rapid maxillary expansion, the case was reassessed, and the patient’s parents opted to have the spaces closed instead of prosthetic restorations. The anterior open bite was solved by closing the gap in the mandibular plane that resulted from the mesial shift of the posterior teeth and by extruding the anterior teeth.

## TREATMENT PROGRESS

**Figure 4:**
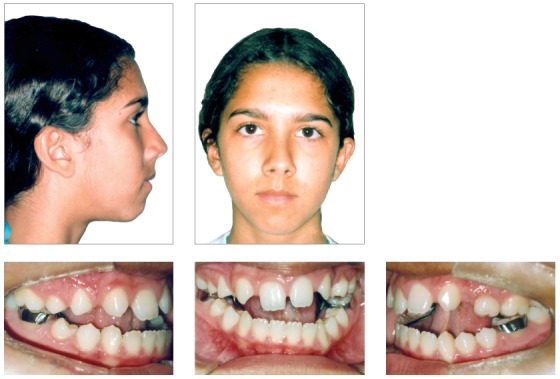
Facial and intraoral photographs after the application of the maxillary expander and vertical chin cup.

**Figure 5:**
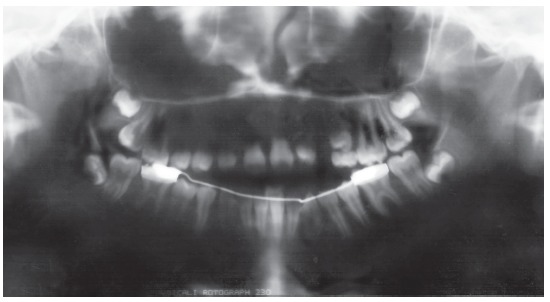
Panoramic radiograph with the lingual arch.

**Figure 6:**
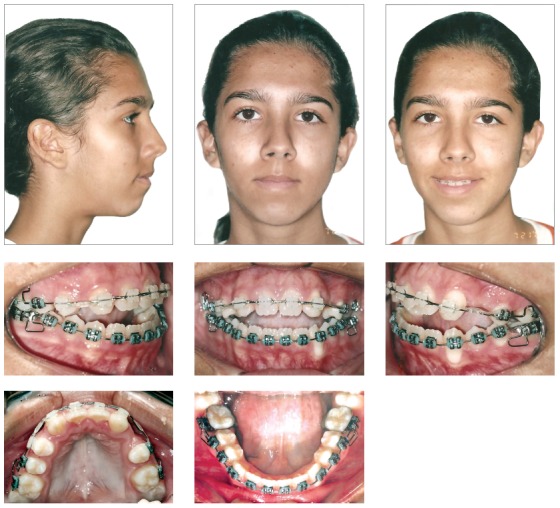
Fixed appliances for the initial alignment and leveling.

**Figure 7:**
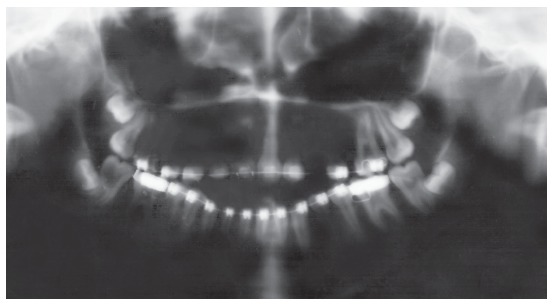
Panoramic radiograph with the fixed appliances.

Initially, the ankylosed upper and lower left deciduous first molars were extracted, and the patient was referred for speech therapy. Then, a cemented encapsulated Haas expander, a lingual arch, and a vertical chincup were placed. The patient was instructed to activate the expander screw in quarter turns once per day for 15 days, at which point the expander remained stable for 6 months (Figs 4 and 5). After 16 months of treatment, the patient was reevaluated. Fixed edgewise appliances were simultaneously placed in the lower and upper arches. Aligning and leveling began with a 0.014-in multi-loop stainless steel wire in the lower arch and 4 x 2 mechanics with 0.014-in stainless steel wire in the upper arch. After eigth months of aligning and leveling, all teeth in the upper arch erupted. A chincup was placed using sky hooks. Using a 0.017 x 0.025-in stainless steel arch with compensating steps for the incisors, the loss of anchorage of the upper posterior teeth began with the use of elastics from the sky hooks to the upper first molars. A new appliance was fixed because the patient requested esthetic braces (Figs 6 and 7). Subsequently, the patient was asked to use anterior elastics. Extractions of the lower third molars were requested. After two years of treatment with the fixed appliance, it was necessary to install one mini-screw between the upper left canine and the second premolar, to aid the loss of anchorage of the left posterior segment. The active treatment time of the second phase was three years and six months, and the complete treatment duration was six years.

When active treatment was completed, the appliances were removed. For retention, the patient was instructed to wear a maxillary wraparound retainer 24h per day, for two years and at night for another six months. In the mandible, a canine-to-canine fixed retainer was bonded.

## TREATMENT RESULTS

**Figure 8:**
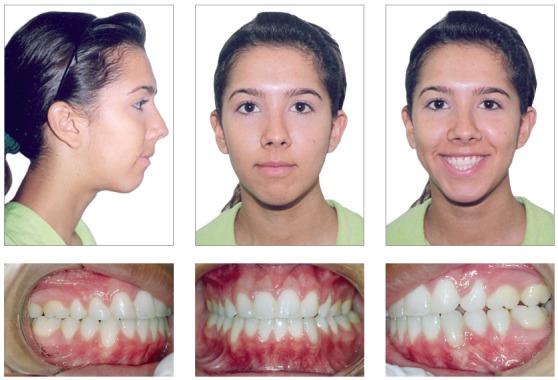
Posttreatment facial and intraoral photographs.

**Figure 9:**
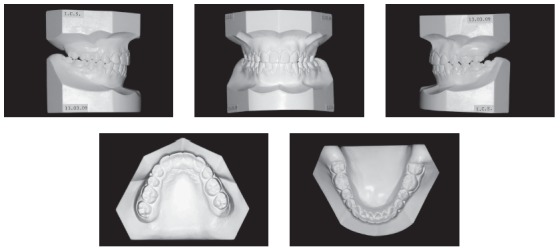
Posttreatment study casts.

**Figure 10:**
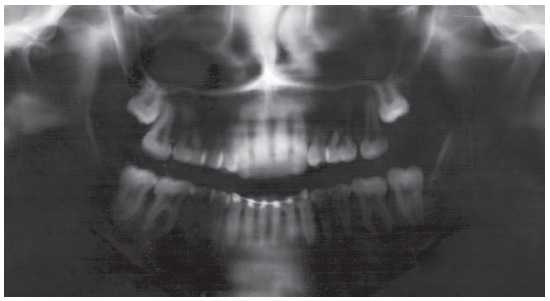
Posttreatment panoramic radiograph.

**Figure 11:**
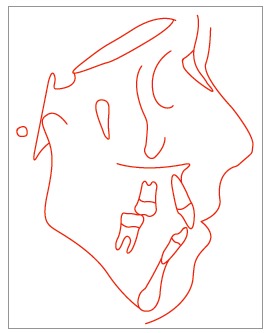
Posttreatment cephalometric tracing.

**Figure 12:**
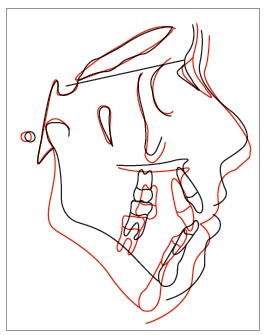
Cephalometric superimposition.

The skeletal pattern of the patient and the choice of a nonsurgical approach in combination produced excellent results in terms of facial appearance and the occlusal relationship (Figs 8-12).

The chincup therapy combined with intermaxillary elastics mechanics and mesial shift of the posterior teeth camouflaged the vertical skeletal problem. The open bite was closed, and good intercuspation was achieved. This was accomplished as a result of the patient’s compliance with the therapy. Minimal root resorption of the teeth was observed even after six years of treatment (Fig 10).

Cephalometric measurements showed that the inherited vertical growth pattern of the mandible was maintained, and the palatal plane rotated clockwise, reducing the anterior open bite. There was a visible improvement in the mandibular teeth and the relationship between upper and lower incisors. The profile became straight, and the patient obtained an appropriated lip seal (Figs 11 and 12).

**Figure 13:**
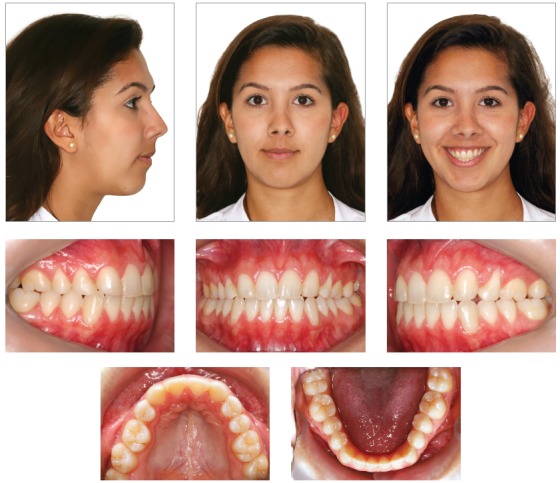
Four-year posttreatment facial and intraoral photographs.

After four years of follow-up, the stability was satisfactory and the horizontal and vertical overlaps established after the treatment were maintained. There was no tooth wear or open spaces, which resulted in excellent stability and the maintenance of periodontal health without relapse. In the retention period, the lingual retainer was removed at the request of the patient (Fig 13).

However, it is important to note that the selection of this treatment resulted in a large asymmetry in the upper dental arch with non-coincident midlines because no replacements for the absent teeth were used. If the option of placing an implant in the upper left first premolar region had been selected, the upper arch would have a Class II relationship.

Despite the satisfaction of the patient after the treatment, she still presenting mandibular retrognathia and vertical maxillary excess.

**Table 1:** Cephalometric analysis.

	Measurements	Normal	A	B
Skeletal pattern	SNA	82°	72°	70°
	SNB	80°	73°	70°
	ANB	2°	1°	0°
	Wits	0 ± 2mm	-3 mm	-2mm
	Angle of convexity	0°	4°	2°
	Y-axis	59°	70°	73°
	Facial angle	87°	80°	77°
	SN-GoGn	32°	48°	53°
Dental pattern	1-NA (degrees)	22°	22°	24°
	1-NA (mm)	4 mm	8°	8°
	1-NB (degrees)	25°	30°	27°
	1-NB (mm)	4 mm	8°	7°
	- Interincisal angle	130°	127°	129°
Profile	Upper lip - S-line	0 mm	2 mm	-2 mm
	Lower lip - S-line	0 mm	2 mm	0°

## DISCUSSION

In this case report, the patient had an open bite with tongue posture problems and excessive vertical growth. Although severe anterior bite is frequently corrected with a combination of orthodontics and orthognathic surgery, many patients reject it due to its seeming aggressive nature, morbidity, and expense.

Orthodontic treatment of patients with skeletal open bite consists of intruding the posterior teeth or preventing further eruption to control the anterior facial height. Several approaches, such as the use of high-pull headgear, chincup therapy, and TADs, have been proposed to achieve improved vertical control.^16^

This patient was treated using a traditional edgewise appliance and chincup combined with intermaxillary elastics. This treatment modality was selected because the patient refused to undergo any invasive treatment. Her 4-year follow-up records show the success of the mechanics without surgery. For this treatment modality, the patient’s motivation and compliance along with the use of the appliances were important.^16^

In the first phase of treatment, an open bite improvement was expected with the vertical-pull chincup used to control vertical growth,^16^ ^-^ ^19^ although this improvement did not occur. This result might be explained by the concomitant use of a maxillary expander. However, the chincup may have prevented molar extrusion.

Despite the positive discrepancy in the arch, disjunction of the palatine suture was required to correct the maxillary atresia that was possibly caused by the agenesis of both the upper lateral incisors and the premolar. This situation seems to imply a paradox: More space was created in the arch with a positive discrepancy. However, this approach allowed for a more favorable shape of the maxilla, which was thought to be essential for a good outcome.

The facial profile was not considered ideal; however, the patient had no esthetic complaints. The patient did not consent to surgery. Despite the complexity of the treatment and the questionable stability of the orthodontics used in this case, the correction of this patient’s functional and morphological problems could only be achieved by camouflage.^19^ ^,^ ^22^

When evaluating alternatives for the correction of agenesis of the lateral incisors, one possible strategy that can be considered is to open the space for implant placement^8^ ^,^ ^9^. However, because of the patient’s young age, even after orthodontic treatment, it would have been necessary to wait for dental-skeletal maturation to implant provisional crowns. Furthermore, a systematic review concluded that tooth-supported dental prosthesis for maxillary lateral incisor agenesis had worse scores in the periodontal indexes compared with an orthodontic space closure.^23^

Another possibility was autogenous tooth transplantation, i.e., transplantation of the lower premolars to the region of the upper lateral incisors. Although the patient’s premolars were at the optimum root development stage for autogenous tooth transplantation, there was not a severe need for space in the lower arch, and there was no need for retraction of the incisors; therefore, there was no indication for the tooth extraction needed for this procedure.^10^ ^,^ ^11^ The upper permanent canines had already been displaced into more mesial positions in the arch space; thus, closure was performed and the canines were reshaped into upper lateral incisors such that the molars would occlude with a Class II relationship.^8^ ^,^ ^12^ ^-^ ^14^

Mesial movement of the upper arch was achieved with the use of elastics from the first permanent molars to the sky hooks that were positioned in a chincup, and this process required strict compliance from the patient. This mesial movement of the posterior teeth helped to decrease the mandibular plane to close the bite. During the mesial shift, we used bendable stainless steel wires with boot-loops to close the bite with the extrusion force component of the anterior teeth. Nickel-titanium or beta-titanium alloys would not have allowed for such control, so these mechanisms were excluded.^24-26^

Due to the agenesis of the left first upper premolar, replacement of the canine tooth was necessary. This was achieved through mesial movement of the second premolar to the canine position, which is among the alternatives to implant placement and space closure. With the help of a mini-implant, a Class II molar position on the left side was established.

Despite the unusual occlusal relationship, correct functional guides were achieved with a canine guide on the right side and a functional guide on the left. The upper molar on the left side was positioned to contact the two opposing teeth. The difficulty and challenges of this case should be considered when evaluating the degree of the success of the treatment. The procedure described herein allowed the patient to retain all of her natural teeth and neither required dental implants nor orthognathic surgery.

Because the patient had a low smile line, it was possible to correct the anterior and lateral open bite using a chincup with mesial movement of posterior teeth, compensation bends in the archwires and interocclusal elastics, which resulted in upper and lower incisor extrusions. The result was a smile line that was found to be pleasant by the patient, nearly coincident dental middle lines, and a good lip seal. After six years, it was possible to satisfy the esthetic and functional needs of the patient in a conservative and efficient manner.^12^ ^-^ ^15^

## CONCLUSION

In nonsurgical treatments, orthodontists camouflage skeletal changes to satisfy the esthetic preferences and functional needs of the patient. This option typically requires more time, is more difficult, and it often results in considerably less satisfactory results, compared to surgical treatment.

The case presented was a great challenge; the prognosis of this case was worse than that of a typical case, due to the three instances of agenesis in the upper arch. However, successful treatment of the severe open bite was achieved, and the ultimate result was an atypical but functional occlusion.

We believe the outcome obtained after the use of a wide range of orthodontic techniques, including a chincup, a palatal expander, sky hooks, and skillful manipulations of the stainless steel wires, was a great achievement in esthetic dentistry in terms of both functional and facial esthetics.
